# Detection and Genetic Diversity of Porcine Coronavirus Involved in Diarrhea Outbreaks in Spain

**DOI:** 10.3389/fvets.2021.651999

**Published:** 2021-02-25

**Authors:** Héctor Puente, Héctor Argüello, Óscar Mencía-Ares, Manuel Gómez-García, Pedro Rubio, Ana Carvajal

**Affiliations:** Department of Animal Health, Faculty of Veterinary Medicine, Universidad de León, León, Spain

**Keywords:** swine coronavirus, pig, PEDV, S or Spike gene, INDEL strain

## Abstract

Porcine enteric coronaviruses include some of the most relevant viral pathogens to the swine industry such as porcine epidemic diarrhea virus (PEDV) or porcine transmissible gastroenteritis virus (TGEV) as well as several recently identified virus such as swine enteric coronavirus (SeCoV), porcine deltacoronavirus (PDCoV) or swine enteric alphacoronavirus (SeACoV). The aim of this study is the identification and characterization of enteric coronaviruses on Spanish pig farms between 2017 and 2019. The study was carried out on 106 swine farms with diarrhea outbreaks where a viral etiology was suspected by using two duplex RT-PCRs developed for the detection of porcine enteric coronaviruses. PEDV was the only coronavirus detected in our research (38.7% positive outbreaks, 41 out of 106) and neither TGEV, SeCoV, PDCoV nor SeACoV were detected in any of the samples. The complete S-gene of all the PEDV isolates recovered were obtained and compared to PEDV and SeCoV sequences available in GenBank. The phylogenetic tree showed that only PEDV of the INDEL 2 or G1b genogroup has circulated in Spain between 2017 and 2019. Three different variants were detected, the recombinant PEDV-SeCoV being the most widespread. These results show that PEDV is a relevant cause of enteric disorders in pigs in Spain while new emerging coronavirus have not been detected so far. However, the monitoring of these virus is advisable to curtail their emergence and spread.

## Introduction

Coronaviruses (CoVs) are found in a wide variety of animals including both mammals and birds in which they cause a variety of respiratory, enteric or even hepatic and neurological disorders (1). They belong to the Coronaviridae family which recognizes four genera based on phylogenetic clustering: Alphacoronavirus, Betacoronavirus, Gammacoronavirus, and Deltacoronavirus. The CoVs are enveloped viruses, and their genome is composed of a non-segmented, positive sense and single-stranded RNA with a size of ~30 kb. From the 5′ end to the 3′ end, their genomic structure is made up of at least six open reading frames (ORFs) named ORF1a, ORF1b, spike (S), envelope (E), membrane (M), and nucleocapsid (N). ORF1a and ORF1b encode non-structural polyproteins, whereas S, E, M, and N genes encode the corresponding structural proteins (2).

Two species of the Alphacoronavirus genus, transmissible gastroenteritis virus (TGEV) and the porcine epidemic diarrhea virus (PEDV) have long been recognized as the cause of acute diarrhea outbreaks on swine farms affecting pigs of all ages and causing high mortality in lactating piglets. The relevance of TGEV on farms is scarce (1), mainly due to the widespread distribution of a respiratory mutant of TGEV, the porcine respiratory coronavirus (PRCV), which partially protects animals against the enteric disease. PEDV was recognized for the first time in Europe and Asia during the seventies and the eighties, respectively (3). In Europe its incidence markedly decreases in the nineties and subsequent years while in Asia PEDV has remained as a major cause of diarrhea outbreaks until now. Moreover, highly pathogenic variants of PEDV have been described in Asia since 2010. This virus emerged in America in 2013–2014 causing substantial economic losses (4). PEDV also re-emerged in Europe soon after its first description in the USA and PEDV outbreaks have been reported in several European countries since 2014 (5–7). Two main PEDV genogroups, named INDEL or G1 and non-INDEL or G2 have been described and differentiated by insertions-deletions in the S1 subunit of the S-gene (8, 9). Non-INDEL isolates have been associated with a higher virulence and better horizontal transmission (10). Both genotypes are reported on infected farms in Asia and America, while in Europe there is no evidence of the presence of the non-INDEL genogroup with the only exception of an Ukrainian isolate (11).

New coronaviruses affecting pigs have been unveiled in recent years. A chimeric virus produced by the recombination of TGEV/PRCV (backbone sequence) with PEDV (S gene), called swine enteric coronavirus (SeCoV), was reported in several European countries including Spain (12–15). A porcine deltacoronavirus (PDCoV) was detected in 2012 in Hong Kong (16) and subsequently on pig farms from the USA, Canada and several Asian countries (17). And finally, a new Alphacoronavirus named swine enteric alphacoronavirus (SeACoV), also known as swine acute diarrhea syndrome coronavirus (SADS-CoV) or porcine enteric alphacoronavirus (PEAV), was identified as the cause of severe diarrhea in neonatal piglets in China (18–22).

The emergence of these new CoVs and re-emergence of PEDV in Europe, immediately after its disruptive appearance in America, requires studies characterizing these CoVs on pig farms so as to allow for an accurate differential diagnosis of viral diarrhea. This study aims at disclosing the current situation of enteric CoVs in Spain, the largest pig producer in Europe, through the identification and characterization of CoVs in diarrhea outbreaks on Spanish pig farms between 2017 and 2019.

## Materials and Methods

### Samples Collection and Preparation

**Figure 1:**
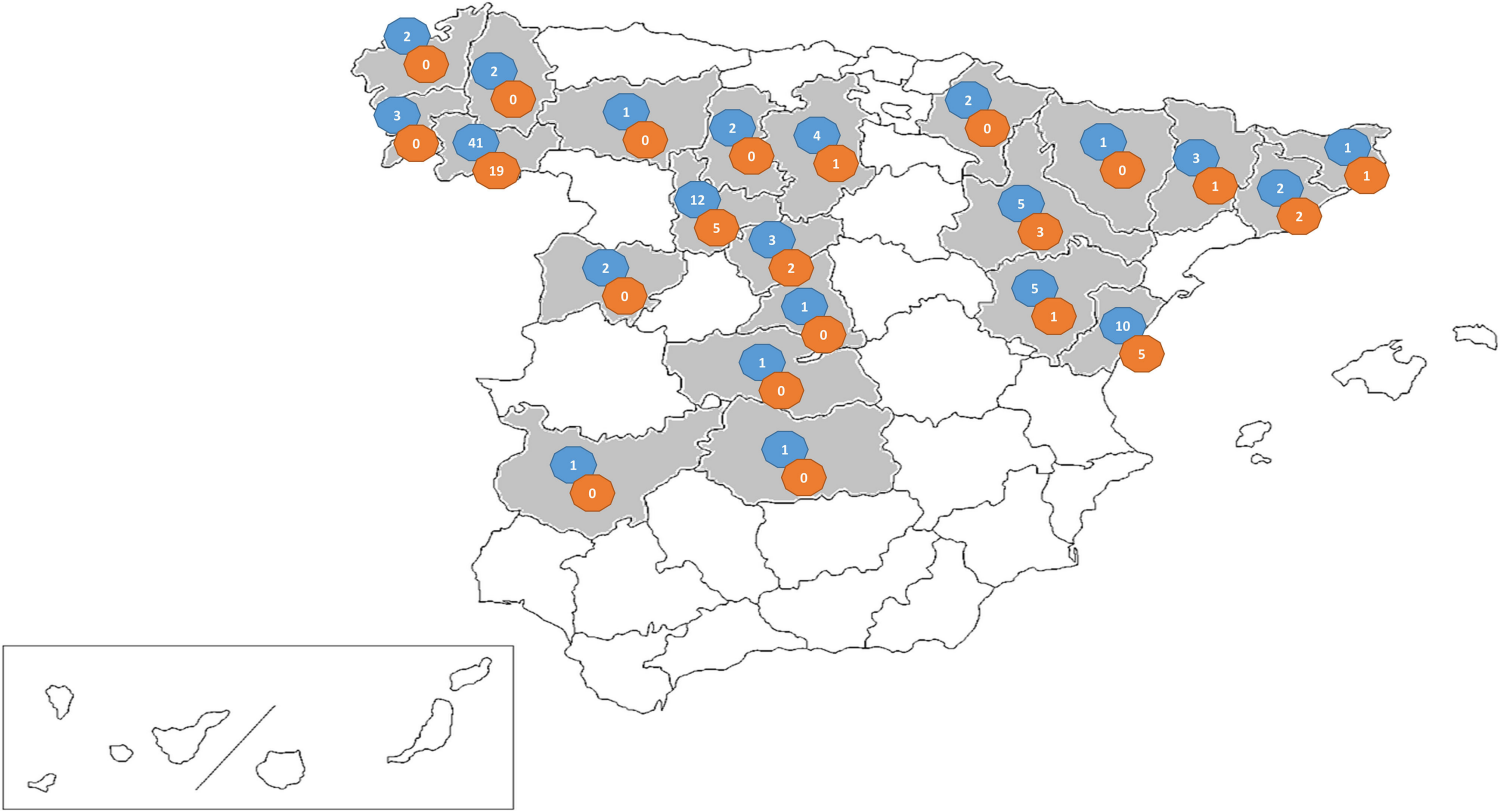
Map showing the distribution of the viral suspected diarrhea outbreaks investigated in this research (shaded area). The number of farms sampled (blue circle) and PEDV positive farms per province (orange circle) are given.

The study was conducted between January 2017 and March 2019 on 106 swine farms (105 from Spain and one from Portugal) with diarrhea outbreaks in which a viral etiology was suspected. The outbreaks affected nursing piglets (<21 days) (28 farms), postweaning-growing pigs (21–70 days) (17 farms), or fattening pigs (>70 days) (61 farms). Location of the farms include 22 provinces in the northeast, center and northwest of Spain (Figure 1). Fecal samples were submitted for diagnostic purposes to the Infectious Diseases Unit of the Animal Health Department of the University of León (Spain). From each farm, two to six individual fecal samples were submitted. Individual fecal samples were mixed to prepare one pooled sample per farm.

Pooled samples were diluted 1:2 (v/v) in sterile phosphate buffered saline (PBS), homogenized by vortex mixing and centrifuged for 10 min at 20,000 g. The RNA was extracted from 140 μl of the supernatant using QIAMP Viral RNA Mini Kit (QIAGEN), following the manufacturer's instructions.

### Molecular Diagnosis of Porcine Enteric Coronaviruses

Two duplex RT-PCRs were developed for the detection of SeACoV (ORF1ab), TGEV/SeCoV (N gene), PEDV/SeCoV (S gene) and PDCoV (N gene) (Table 1). Two conventional RT-PCRs were also developed to confirm SeCoV by excluding a potential PEDV-TGEV co-infection, by detecting the M gene of PEDV and the S gene of TGEV. The RT-PCR was carried out with Verso 1-Step RT-PCR ReddyMix kit (Thermo Scientific). The reaction was conducted under the following conditions: 50°C for 30 min, 95°C for 2 min, 40 cycles at 95°C for 20 s, 50°C for 30 s, and 72°C for 1 min, followed by a final extension step at 72°C for 10 min.

**Table 1:** Primer sets used for the detection of porcine enteric coronaviruses by multiplex RT-PCR and for the amplification of the S gene of PEDV.

**PCR type**	**Viral agent**	**Sequence 5^**′**^ to 3^**′**^**	**Gene target**	**Product size (bp)**	**References**
Multiplex RT-PCR 1	SeACoV	TTTTGGTTCTTACGGGCTGTT	RNA-dependent	754	(20)
		CAAACTGTACGCTGGTCAACT	RNA polymerase		
	TGEV/SeCoV	GATGGCGACCAGATAGAAGT	Nucleoprotein	612	(23)
		GCAATAGGGTTGCTTGTACC			
Multiplex RT-PCR 2	PEDV/SeCoV	TTCTGAGTCACGAACAGCCA	Spike	651	(24)
		CATATGCAGCCTGCTCTGAA			
	PDCoV	GCTGACACTTCTATTAAAC	Nucleoprotein	497	(25)
		TTGACTGTGATTGAGTAG			
Conventional RT-PCR 1	TGEV	GTGGTTTTGGTYRTAAATGC	Spike	858	(24)
		CACTAACCAACGTGGARCTA			
Conventional RT-PCR 2	PEDV	GGGCGCCTGTATAGAGTTTA	Membrane	412	(23)
		AGACCACCAAGAATGTGTCC			
PEDV S-gene RT-PCR	PEDV	TGCTAGTGCGTAATAATGAC	Spike 1	1,349	(26)
		CGTCAGTGCCATGACCAGTG			(15)
		GGGAAATTGTCATCACCAAG	Spike 2	1,289	(15)
		CTGGGTGAGTAATTGTTTACAACG			(27)
		AGTACTAGGGAGTTGCCTGG	Spike 3	1,216	(15)
		AACCATAACGCTGAGATTGC			
		TTGAACACTGTGGCTCATGC	Spike 4	1,128	(15)
		CATCTTTGACAACTGTGT			(26)

The RT-PCR products were visualized on a 1.5% agarose gel containing RedSafe Nucleic Acid Staining Solution (iNtRON Biotechnology, Inc.). The length of the PCR fragment generated for each CoV is shown in Table 1.

### PEDV S-gene Sequencing

The S-gene of PEDV positive samples was amplified using four overlapping fragments with the primers described in Table 1 and the Verso 1-Step RT-PCR ReddyMix kit (Thermo Scientific). The reaction was conducted under the following conditions: 50°C for 30 min, 95°C for 2 min, 45 cycles at 95°C for 20 s, 50°C for 30 s, and 72°C for 2 min, followed by a final extension step at 72°C for 10 min. The RT-PCR products were purified using the GeneMATRIX Basic DNA Purification Kit (EurX). The complete sequences of the S-gene were obtained by using forward and reverse Sanger sequencing. The complete sequence of the S gene of 36 PEDV isolates from different farms can be accessed at the NCBI GenBank with the accession numbers MW251343-MW251378 (Table 2). The remaining five PEDV isolates included in this research were previously sequenced by using a RNA virus-specific tailor-made NGS protocol (15).

**Table 2:** List of porcine epidemic diarrhea virus (PEDV) isolates recovered in this study.

**Isolate name**	**Collection date**	**Country**	**Province of origin**	**Accession number**
SP-VC2^*^	12/01/2017	Spain	Valladolid	MN692784
SP-VC3	17/01/2017	Spain	Valladolid	MW251343
SP-VC4	17/01/2017	Spain	Burgos	MW251344
SP-VC16	01/03/2017	Spain	Zaragoza	MW251345
SP-VC18	07/03/2017	Spain	Segovia	MW251346
SP-VC19	07/03/2017	Spain	Segovia	MW251347
SP-VC27	06/06/2017	Spain	Ourense	MW251348
SP-VC29	06/06/2017	Spain	Ourense	MW251349
SP-VC46	11/01/2018	Spain	Valladolid	MW251350
SP-VC51^*^	02/02/2018	Spain	Ourense	MN692788
SP-VC52	02/02/2018	Spain	Ourense	MW251351
SP-VC53	05/02/2018	Spain	Teruel	MW251352
SP-VT13	14/02/2018	Spain	Valladolid	MW251353
SP-VC54	14/02/2018	Spain	Castellón	MW251354
SP-VC55	15/02/2018	Spain	Girona	MW251355
SP-VC57^*^	02/03/2018	Spain	Castellón	MN692789
SP-VC61	18/04/2018	Spain	Castellón	MW251356
SP-VC62^*^	03/05/2018	Spain	Castellón	MN692790
SP-VC63	03/05/2018	Spain	Zaragoza	MW251357
SP-VC66	30/05/2018	Spain	Ourense	MW251358
SP-VC68	20/06/2018	Spain	Zaragoza	MW251359
SP-VC75	05/09/2018	Spain	Castellón	MW251360
SP-VC77	05/09/2018	Spain	Ourense	MW251361
SP-VC81	06/10/2018	Spain	Ourense	MW251362
SP-VC87	07/10/2018	Spain	Lérida	MW251363
SP-VT86	07/11/2018	Spain	Barcelona	MW251364
SP-VT87	29/11/2018	Spain	Valladolid	MW251365
SP-VT108	10/01/2019	Spain	Barcelona	MW251366
SP-VC89	18/01/2019	Spain	Ourense	MW251367
SP-VC90	22/01/2019	Spain	Ourense	MW251368
SP-VC92	24/01/2019	Spain	Ourense	MW251369
SP-VC93	25/01/2019	Spain	Ourense	MW251370
SP-VC94	08/02/2019	Spain	Ourense	MW251371
SP-VC95	12/02/2019	Spain	Ourense	MW251372
SP-VC96	12/02/2019	Spain	Ourense	MW251373
SP-VC97	14/02/2019	Spain	Ourense	MW251374
SP-VC98	19/02/2019	Spain	Ourense	MW251375
SP-VC99	19/02/2019	Spain	Ourense	MW251376
SP-VC100^*^	28/02/2019	Spain	Ourense	MN692791
SP-VC101	28/02/2019	Spain	Ourense	MW251377
POR-VC102	14/03/2019	Portugal	Coimbra	MW251378

*A complete sequence of the S gene was obtained for all PEDV isolates*.

* *PEDV isolates previously sequenced by de Nova et al. (15)*.

### Phylogenetic Analysis

PEDV and SeCoV genome sequences available in the GenBank database were aligned together with S-gene sequences obtained in this study using CLUSTALW. After the alignment, the evolutionary relationships among sequences were analyzed with a phylogenetic analysis, using the neighbor joining method and the maximum composite likelihood method with MEGAX software (28).

### Statistical Analysis

The Fisher exact test was used to compare the frequency of occurrence of PEDV positive outbreaks among age groups and provinces (only those with five or more submitted samples were included in the analysis). ANOVA test was used to compare the number of investigated outbreaks as well as the percentage of PEDV positive outbreaks among the different trimesters of the year. Epi Info™ was used for data analysis at α = 0.05.

## Results

### Prevalence of Enteric Coronaviruses in Porcine Diarrhea Outbreaks

Neither TGEV, SeCoV, PDCoV nor SeACoV were detected in any of the samples, while PEDV was the only coronavirus detected in 41 out of the 106 investigated outbreaks (38.7%). Most of these outbreaks occurred in fattening pigs (24 positive farms out of 61, 39.3%) or postweaning-growing pigs (nine positive farms out of 17, 52.9%). PEDV was involved to a lesser extent in diarrhea outbreaks affecting nursing piglets (eight positive farms out of 28, 28.6%), although no significant differences in the number of PEDV confirmed outbreaks between age groups arose when compared using the Fisher exact test (*p* = 0.094).

PEDV positive farms were detected throughout the sampled area in northeast, center, and northwest of the country with at least one positive outbreak in 10 out of 22 sampled provinces (Figure 1). No significant differences were found in the number of PEDV confirmed outbreaks between provinces (*p* = 0.286).

**Figure 2:**
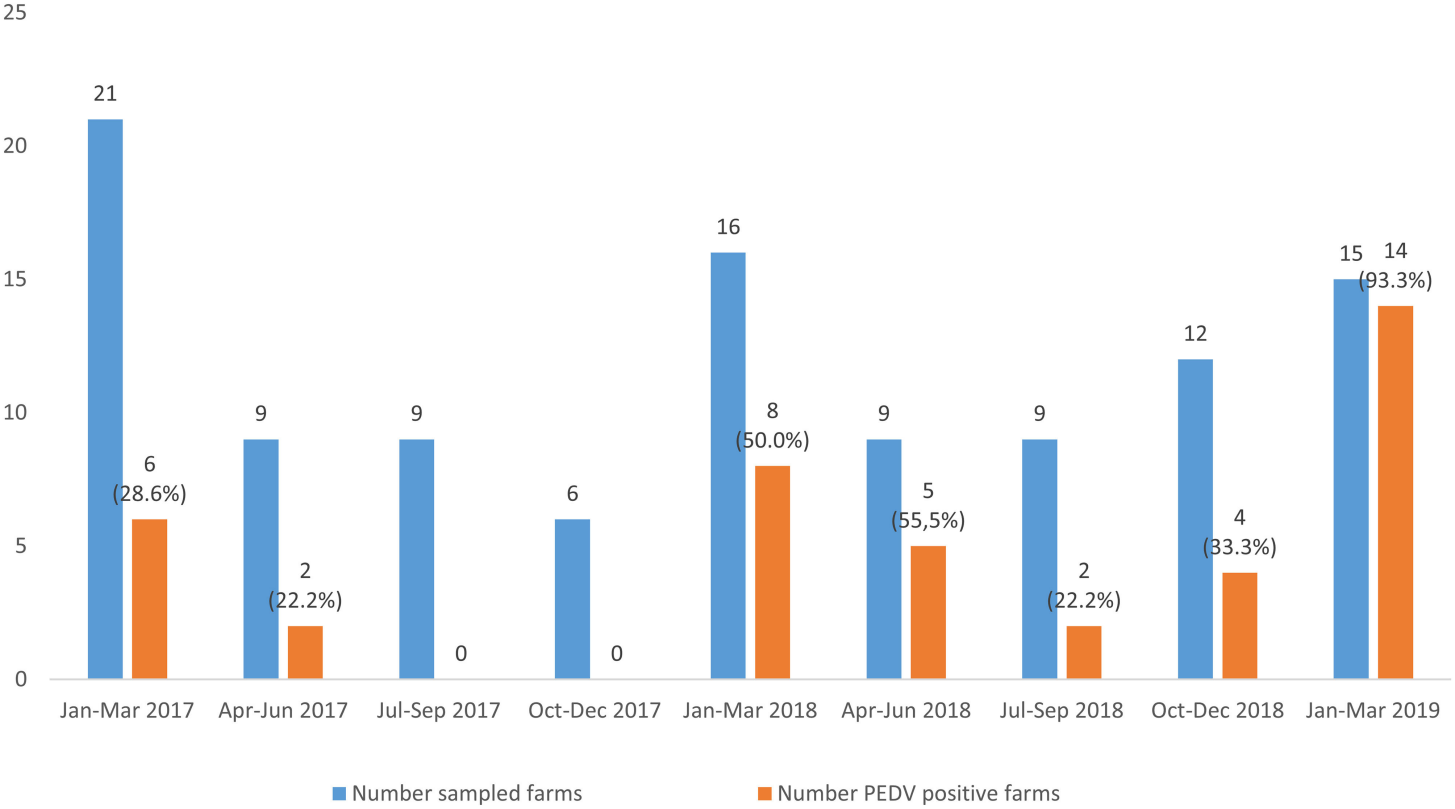
Distribution of the viral suspected diarrhea outbreaks and PEDV positive outbreaks from January, 2017 to March, 2019. The number of sampled farms and PEDV-positive farms is shown above the columns. The percentage of PEDV-positive outbreaks in each trimester studied. The percentage of PEDV-positive outbreaks in each trimester studied is showed.

In addition, it was evidenced that most of the investigated outbreaks occurred during the first trimester of the year (*p* = 0.041) (Figure 2). Although most of the PEDV positive outbreaks also occurred during the first 3 months of the year, no significant differences arose when compared using ANOVA test (*p* = 0.097).

### Phylogenetic Analysis Based on the Nucleotide and Amino Acid Sequences of PEDV S Gene

**Figure 3:**
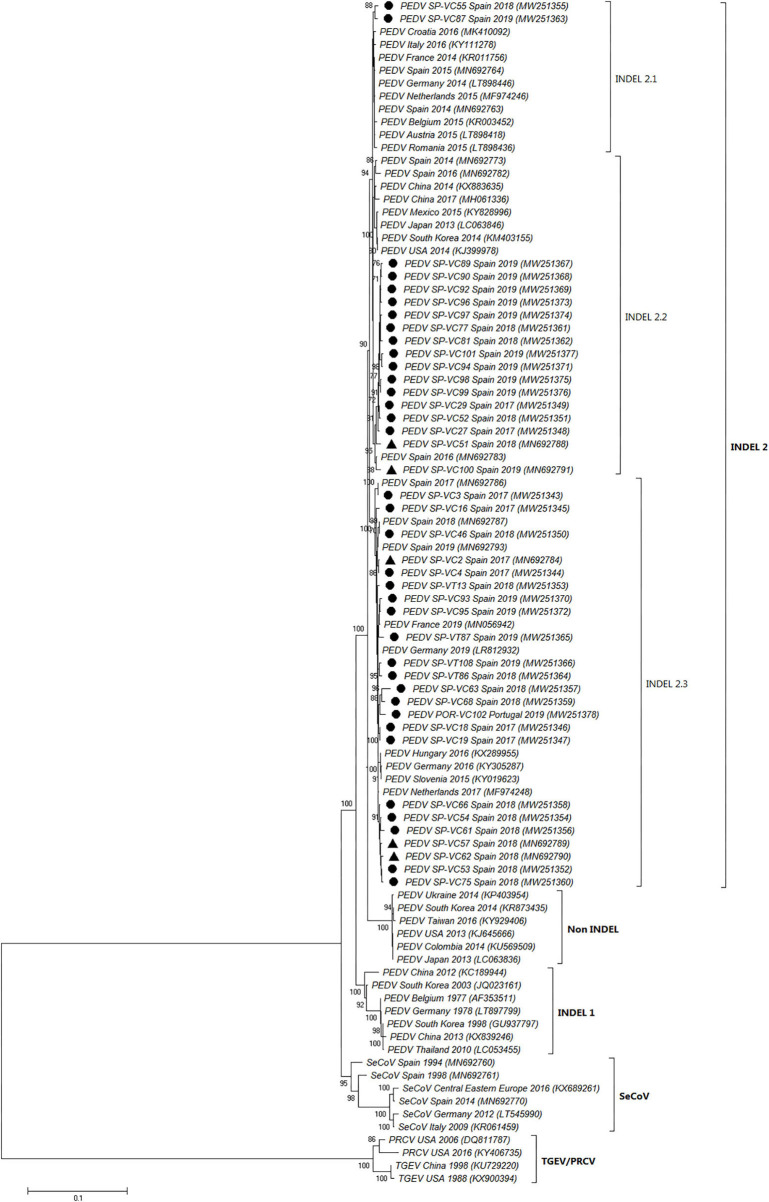
Phylogenetic tree based on the complete S-gene sequences including enteric porcine coronaviruses available in GenBank. Numbers along the tree represent the confidence value for a given internal branch based on 500 Bootstrap replicates (only values >70 are shown). The symbols above the strains highlight the Spanish PEDV isolates of this study. The filled circles identified the isolates sequenced in this research while the filled triangles identified isolates previously sequenced by de Nova et al. (15). GenBank accession number, country and year of the outbreak are also shown below the strains. The genogroups and subgroups referred in the text are included on the right of the tree. Scale bars indicate nucleotide substitutions per site.

The full-length S-gene sequences of all PEDV positive samples (41) were compared to previous and recent sequences of PEDV and SeCoV available in GenBank (Figure 3, Supplementary Data 1). The phylogenetic tree showed that all PEDV isolates recovered in Spain between 2017 and 2019 were allocated within the INDEL 2 or G1b genogroup together with several recent European PEDV isolates and isolates from the USA and Asia. They clustered in a branch clearly separate from the non-INDEL or G2 genogroup as well as from the original European or Asian PEDV isolates included in the INDEL 1 or G1a genogroup and SeCoV isolates.

**Figure 4:**
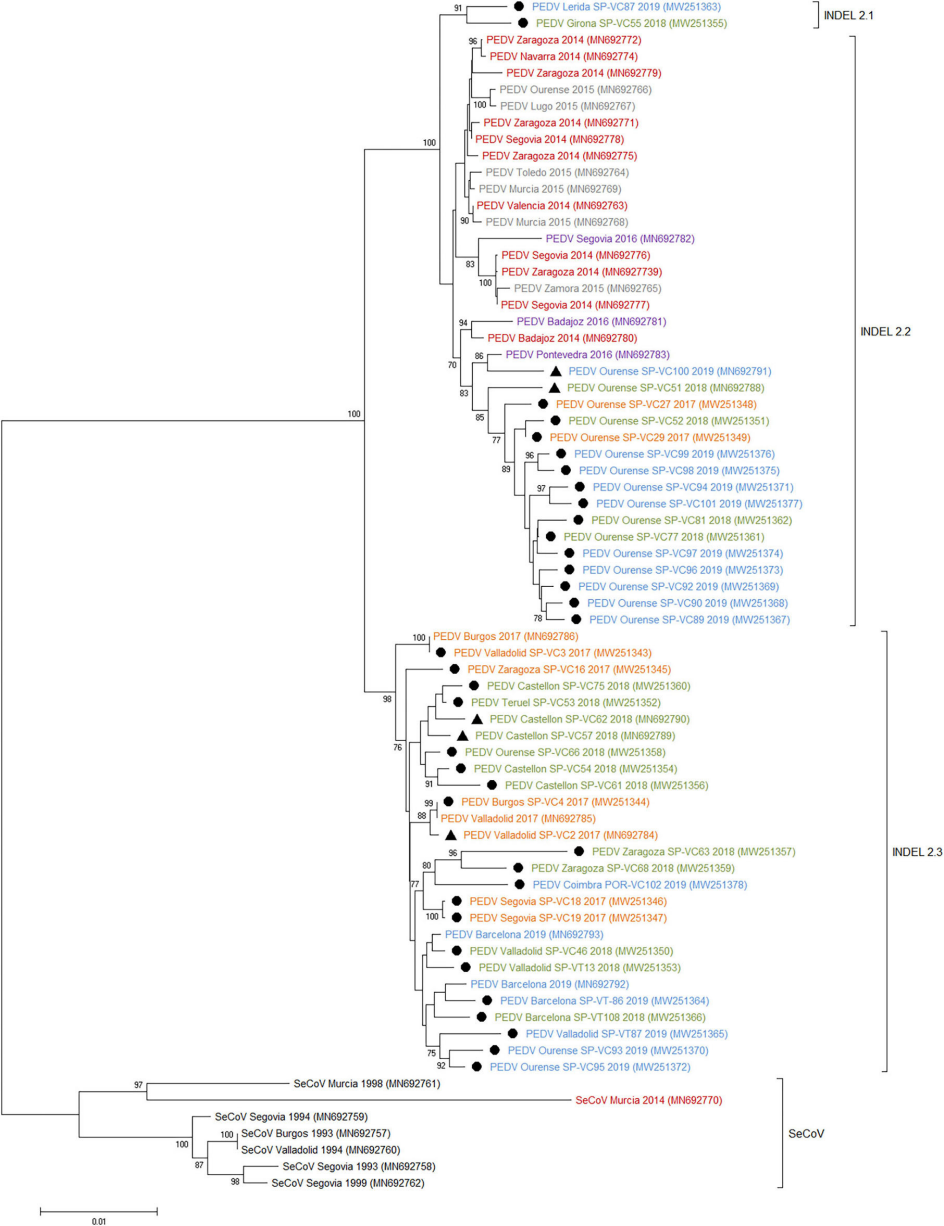
Phylogenetic tree based on the complete S-gene sequences including all Spanish enteric porcine coronaviruses available in GenBank. Numbers along the tree represents the confidence value for a given internal branch based on 500 Bootstrap replicates (only values >70 are shown). The symbols above the strains stand out the Spanish PEDV isolates of this study. The filled circles identified the isolates sequenced in this research and the filled triangles identified the isolates previously sequenced by de Nova et al. (15). GenBank accession number, province and year of the outbreak are also shown below the strains. The genogroups and subgroups referred in the text are included on the right of the tree. Scale bars indicate nucleotide substitutions per site. Black, pre-2000 isolates; Red, 2014 isolates; Gray, 2015 isolates; Purple, 2016 isolates; Yellow, 2017 isolates; Green, 2018 isolates; Blue, 2019 isolates.

Three subgroups or clusters were identified from Spanish PEDV isolates identified as INDEL 2.1, 2.2, and 2.3 (Figures 3, 4). The first was formed using two Spanish isolates recovered in 2018 and 2019 together with other Spanish and European PEDV isolates from 2014 to 2016. The INDEL 2.2 cluster included Spanish isolates from 2014 to 2019 and several Asian and American PEDV strains from the same time period. It is worth mentioning that all the isolates identified in this research included in the INDEL 2.2 subgroup correspond to isolates recovered from farms located within the same region and belong to the same pig producing company. Finally, the INDEL 2.3 cluster include recent Spanish isolates from 2017 to 2019 distributed throughout the sampling area together with Hungarian, Slovenian, Dutch, German, and French coetaneous strains. This clade corresponds to a PEDV-SeCoV recombinant variant previously described in Hungary and Slovenia (29, 30) as well as in Spain (15).

Three major regions of the S1 gene were further analyzed and characterized at the amino acid level. Compared with three different strains of PEDV (CV777 accession no. AF353511, OH851 accession no. KJ399978 and SLOreBAS-1 accession no. KY019623), four amino acid mutations were found in four out of 41 Spanish PEDV isolates (Supplementary Data 1).

## Discussion

The recent emergence of several novel porcine enteric coronaviruses such as PDCoV or SeACoV together with the re-emergence of PEDV, a classical coronavirus, and their spread throughout the main pig producing countries over the last years have highlight porcine enteric coronavirus. They have caused significant losses to the pig-farming industry associated to high morbidity acute diarrhea in pigs of all ages and high mortality in neonatal pigs in naive farms. Enteric diseases caused by porcine enteric CoVs are clinically indistinguishable thus making differential diagnosis in the laboratory an essential tool.

A hundred and six viral suspected diarrhea outbreaks were investigated between 2017 and 2019 and confirmed that PEDV was the only enteric coronavirus detected in swine farms in Spain. PEDV was identified in about a 40% of the investigated outbreaks, confirming the re-emergence of PEDV in the Iberian Peninsula as it has been described in several European countries (31) after its emergence in 2013 in the United States. The difference in the percentage of PEDV positive outbreaks among the different trimesters of the year was near to statistical significance, with most of the PEDV positive outbreaks occurring in winter (68.3%, 28 out of 41 PEDV positive outbreaks), when low temperatures favor the environmental resistance of the virus facilitating its indirect transmission. This results confirms the seasonal distribution of the disease (3, 32). Besides, although no significant differences were shown in the proportion of PEDV outbreaks between age groups, most of the outbreaks were observed among postweaning-growing or fattening pigs. The fact that few PEDV outbreaks were detected in suckling piglets (28.6%, eight PEDV outbreaks out of 28 investigated) could be a consequence of maternal immunity in the sows or high biosecurity level in farrowing facilities. Clearance of maternal antibodies together with the mix of piglets after weaning could explain a higher percentage of positive outbreaks (52.9%, nine PEDV positive outbreaks out of 17 investigated) in postweaning pigs (21–70 days) (1).

Neither TGEV, SeCoV, PDCoV nor SeACoV were detected in any of the investigated diarrhea outbreaks. To our knowledge, this is the first study in Europe actively researching PDCoV or SeACoV on swine farms. While SeACoV has a limited geographic distribution and has only been detected in swine farms in China (18, 19), PDCoV has been reported in the USA, South Korea, Thailand and mainland China (33). Although the country of origin and transmission routes of this virus have not been elucidated, available sequence data suggests that PDCoV identified in South Korea was introduced from the USA (33) indicating its international spread. The ability of porcine CoVs to emerge and re-emerge showed in recent years together with the probable naive status of European pig population for these emerging CoVs allows us to conclude the need of monitoring programmes which allow for a prompt detection and alert to establish strict biosecurity measures which would curtail their spread between countries or continents.

In order to identify PEDV variants currently circulating in Spain, we obtained the complete sequences of the S-gene of all the isolates and compared them to a representative selection of 44 PEDV genome sequences from Europe, Asia and America available in the GenBank (Figure 3). Like in other European countries (5–7, 32), only PEDV isolates included in the INDEL 2 or G1b genogroup were identified in Spain between 2017 and 2019. This genogroup has been described as causing a less severe disease than the non-INDEL or G2 genogroup (3, 8) which could explained the limited economic impact of PEDV re-emergence in Europe as compared to its dramatic consequences in the United States or Asia (34).

Phylogenetic analysis allows us to classify recent Spanish PEDV isolates into three clusters with some geographical relationships. The INDEL 2.1 and 2.2 clusters have a limited geographical spread, with the first restricted to two isolates recovered from two farms from Catalonia in the northeast of the country and the second including a number of isolates from farms located in a single province in the northwest and belonging to the same pig-producing company. Nevertheless, the INDEL 2.3 cluster included isolates recovered throughout the country together with recombinant PEDV-SeCoV isolates described in Hungary, Slovenia, Italy, or Spain (14, 15, 29) as well as Dutch, German, and French PEDV recent isolates. These isolates harbor a recombinant fragment of ~400 nt in the 5′ end of S-gene with PEDV and SeCoV being the major and minor parents, respectively. Since S protein is a key target for PEDV neutralizing antibodies, it has been proposed that this recombination event might provide some advantages (35, 36). Our results confirm this recombinant PEDV-SeCoV variant as the most widespread in Spain between 2017 and 2019 as previously suggested by de Nova et al. (15) in a research with a limited number of recent PEDV isolates. A similar displacement of other PEDV subgroups by the PEDV-SeCoV cluster has also been reported in Italy (35).

To sum up, this research confirms that PEDV has become endemic in Spain being detected in almost 40% of the viral suspected diarrhea outbreaks between 2017 and 2019 with most of the outbreaks occurring in postweaning-growing or fattening pigs, which limits the severity of the disease. Only the PEDV INDEL 2 or G1b genogroup is circulating and the recombinant PEDV-SeCoV variant is the most widespread strain. In contrast, neither PEDV non-INDEL or G2 genogroup, nor TGEV, SeCoV, PDCoV or SeACoV were detected. The emergence of new virus or variants due to spillover or through mutation or recombination events makes monitoring of porcine enteric CoVs of outmost importance in order to prevent their spread and allow for updated diagnostic tools.

## Data Availability Statement

The datasets presented in this study can be found in online repositories. The names of the repository/repositories and accession number(s) can be found in the article/Supplementary Materials.

## Author Contributions

HP, HA, PR, and AC conceived and designed the experiments, analyzed the data, and wrote and revised the manuscript. HP, MG-G, and ÓM-A performed the experiments. All authors contributed to the article and approved the submitted version.

## Conflict of Interest

The authors declare that the research was conducted in the absence of any commercial or financial relationships that could be construed as a potential conflict of interest.

## Supplementary Material

Supplementary Data 1 Comparison of S1 amino acid sequences of PEDV isolates from this study with those of reference strains (GenBank accession nos. AF353511, KJ399978, and KY019623). Amino acid sequence variations in three major epitope regions (aa 220-233, aa 770-776, and aa 784-792) in the S1 genes of 41 Spanish PEDV strains.
